# Supplementary material for: A dynamic mode of mitotic bookmarking by transcription factors
Source: eLife. 2016 Nov 19;5:e22280. doi: 10.7554/eLife.22280 (PMC5156526; doi:10.7554/eLife.22280)
Supplement: Source code 1. — DOI: http://dx.doi.org/10.7554/eLife.22280.022 [file elife-22280-code1.zip › SLIMfast/Manuel SLIMfast.docx]

**Manuel SLIMfast**

1. Lancer SLIMfast depuis le terminal MATLAB.


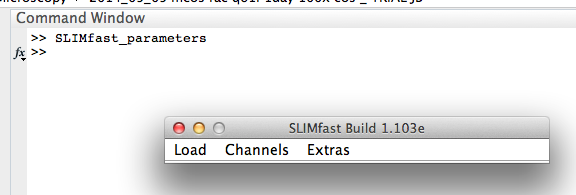


1. Charger un film en format TIFF depuis le menu “Load”.


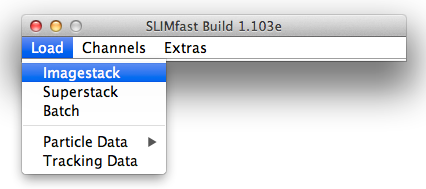


3. Selectionner le menu “OPT” sur le paneau du film.


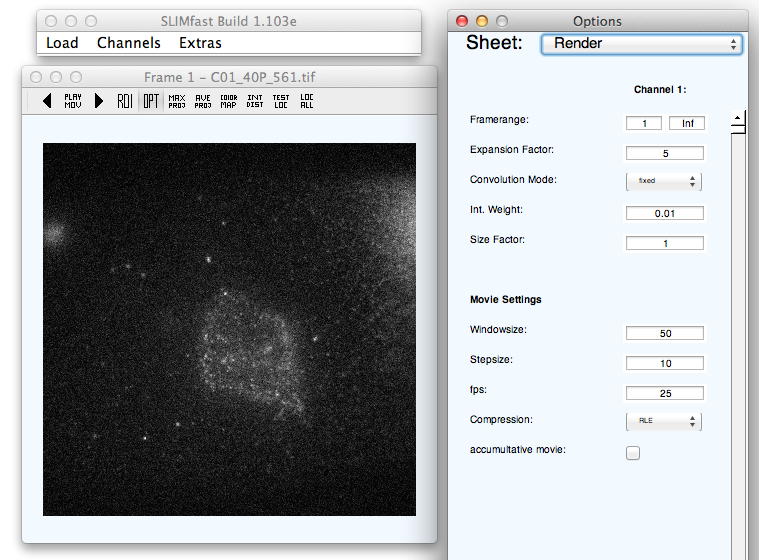


4. Dans la fenêtre “Options”, selectionner le Sheet “Acquisition”, et préciser les champs “Pixel Size” et “Lag Time” (temps d’exposition).


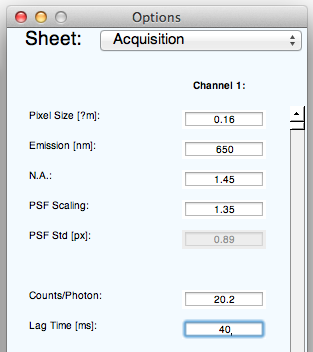


5. Dans la fenêtre du film, appuer sur “LOC ALL”. Un fenêtre de progrès va apparaitre.

Ici on presume que les paramètres de localization sont raisonnables, sinon, on pourrait les changer sous le Sheet “Localization”. D’ailleurs, on peut tester la qualité de localisation en appuyant sur “TEST LOC” dans la fenêtre du film.


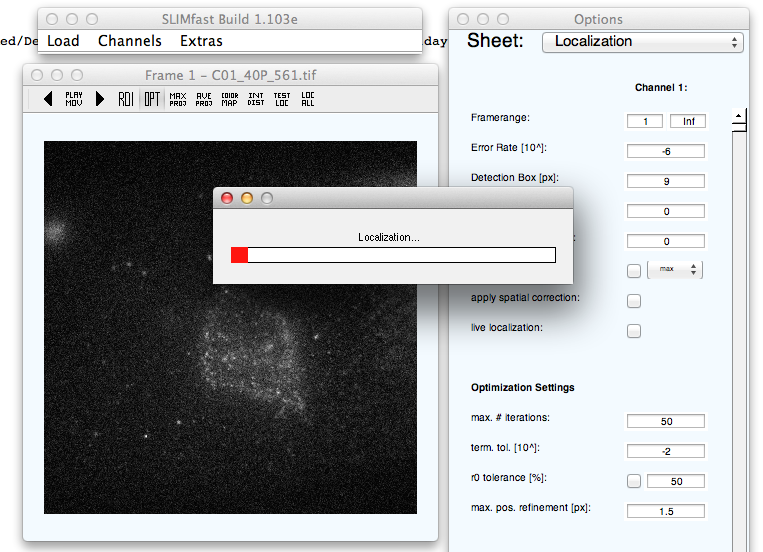


6. Après que la localization soit fait, sélectionner “SLIMfast” sous “Particle Data” dans la menu “Load”, et sélectionner le fichier .mat qui vient d’être créer. Une image de tous les localization sera affichée.


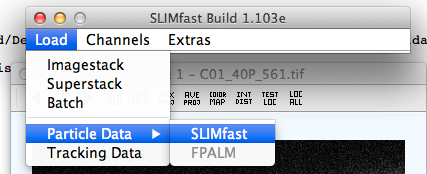


7. Selectionner “OPT” dans la nouvelle fenêtre et préciser les paramètres de tracking dans le Sheet “Tracking” (les parametres de défaut suffisent dans la plupart des cas).


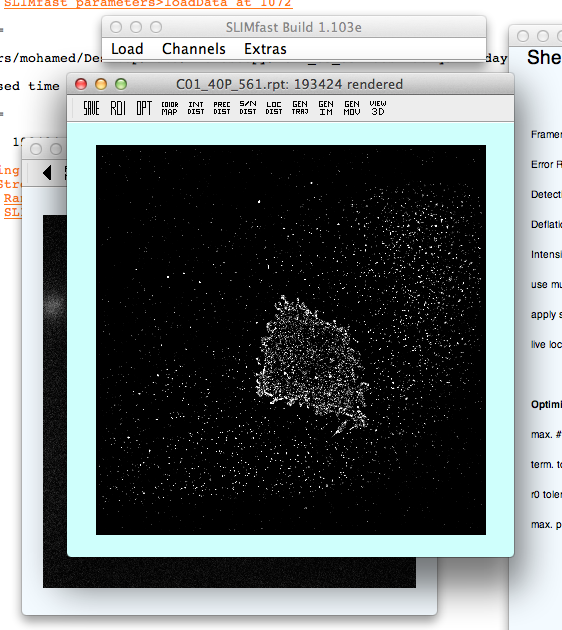

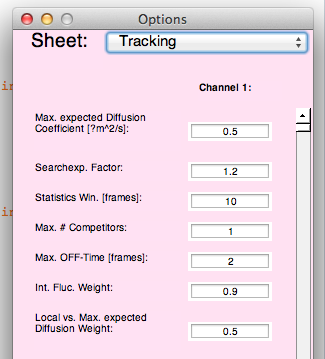


8. Appuyer sur “GEN TRAJ” afin d’effectuer le tracking.


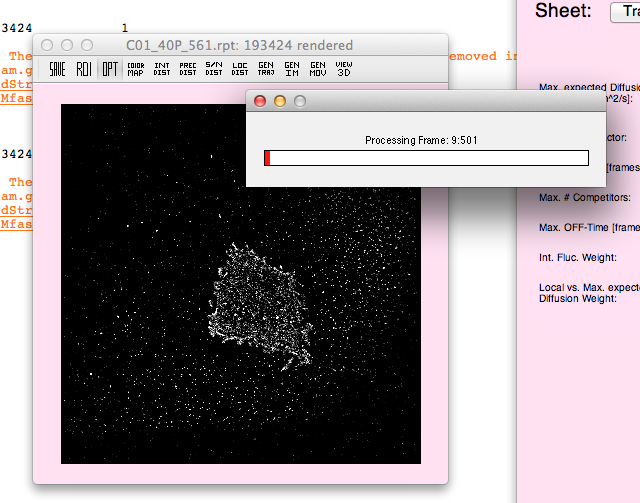


9. Une fois que les trajectoires sont crées, ouvrir le fichier MATLAB “evalSPT_3”
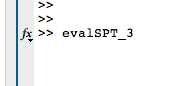


10. Appuyer sur le bouton “+” pour charger votre fichier trajectoire créé (un fichier qui termine en rpt_tracked.mat), et appuyer sur “OK”.
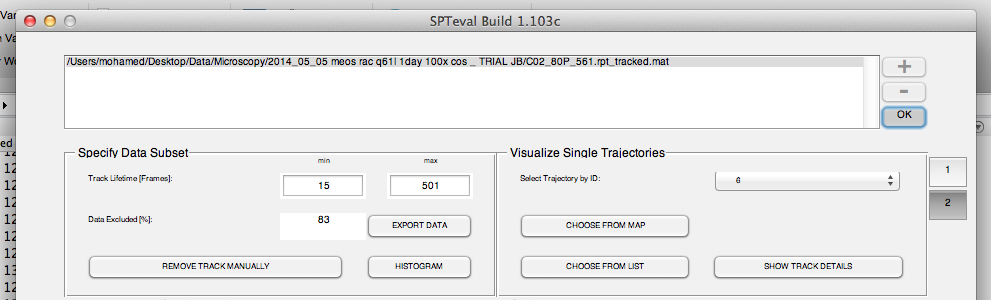


11. Ici on sélectionne la durée minimum et maximum de les trajectoires qu’on veut exporter (dans le paneau “Specify Data Subset”).


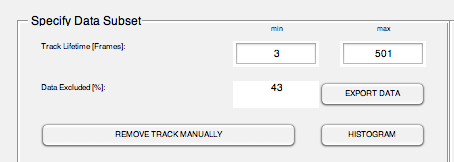


12. Appuyer sur “EXPORT DATA” pour sauvegarder vos trajectoires en format .txt (ASCII). Les colonnes pertinentes dans le fichier sont:

colonne 1: coordonnée x (en pixel)

colonne 2: coordonnée y (en pixel)

colonne 3: numéro de l’image dans le film

colonne 4: numéro de la trajectoire
